# The causality between gut microbiome and liver cirrhosis: a bi-directional two-sample Mendelian randomization analysis

**DOI:** 10.3389/fmicb.2023.1256874

**Published:** 2023-10-18

**Authors:** Qing-Ao Xiao, Yun-Fei Yang, Lin Chen, Ying-Chun Xie, Hai-Tao Li, Zhi-Gang Fu, Qiang Han, Jia Qin, Jie Tian, Wen-Jiang Zhao, Fei Cai, Yin-Tao Hu, Lin-Feng Ai, Chao Li, Xu-Ying Chen, Decheng Wang, Yu-Yan Tan, Xuan Xia, Xiao-Lin Zhang

**Affiliations:** ^1^Department of Interventional Radiology, The First College of Clinical Medical Science, China Three Gorges University, Yichang, China; ^2^Yichang Central People's Hospital, Yichang, China; ^3^Department of Surgery of Thyroid and Breast, The First College of Clinical Medical Science, China Three Gorges University, Yichang, China; ^4^Department of Radiology, The First College of Clinical Medical Science, China Three Gorges University, Yichang, China; ^5^Hubei Key Laboratory of Tumor Microenvironment and Immunotherapy, College of Basic Medical Sciences, China Three Gorges University, Yichang, China; ^6^Institute of Infection and Inflammation, China Three Gorges University, Yichang, China; ^7^Department of Physiology and Pathophysiology, College of Medical School, China Three Gorges University, Yichang, Hubei, China

**Keywords:** gut microbiome, Mendelian randomization, cirrhosis, primary biliary cirrhosis, alcoholic cirrhosis

## Abstract

**Background and aim:**

Previous studies have reported an association between gut microbiota and cirrhosis. However, the causality between intestinal flora and liver cirrhosis still remains unclear. In this study, bi-directional Mendelian randomization (MR) analysis was used to ascertain the potential causal effect between gut microbes and cirrhosis.

**Methods:**

Large-scale Genome Wide Association Study (GWAS) data of cirrhosis and gut microbes were obtained from FinnGen, Mibiogen consortium, and a GWAS meta-analysis of Alcoholic cirrhosis (ALC). Two-sample MR was performed to determine the causal relationship between gut microbiota and cirrhosis. Furthermore, a bi-directional MR analysis was employed to examine the direction of the causal relations.

**Result:**

In MR analysis, we found that 21 gut microbiotas were potentially associated with cirrhosis. In reverse MR analysis, 11 gut microbiotas displayed potentially associations between genetic liability in the gut microbiome and cirrhosis. We found that the family *Lachnospiraceae* (OR: 1.59, 95% CI:1.10–2.29) might be harmful in cirrhotic conditions (ICD-10: K74). Furthermore, the genus *Erysipelatoclostridium* might be a protective factor for cirrhosis (OR:0.55, 95% CI:0.34–0.88) and PBC (OR:0.68, 95% CI:0.52–0.89). Combining the results from the MR analysis and reverse MR analysis, we firstly identified the *Genus Butyricicoccus* had a bi-directional causal effect on PBC (Forward: OR: 0.37, 95% CI:0.15–0.93; Reverse: OR: 1.03, 95% CI:1.00–1.05).

**Conclusion:**

We found a new potential causal effect between cirrhosis and intestinal flora and provided new insights into the role of gut microbiota in the pathological progression of liver cirrhosis.

## Graphical Abstract

In this study, we employed Mendelian randomization method to investigate the causal correlation between gut microbiota and liver cirrhosis. We offered a novel perspective to explore the association between gut microbiota and the development of liver cirrhosis. Our findings revealed 21 specific gut microbiota that are significantly associated with the onset of cirrhosis, including the microbiota that have not been previously reported in literatures. Furthermore, our reverse analysis demonstrated that the state of liver cirrhosis influences the abundance of 11 distinct types of intestinal flora which have not been partly reported before. 
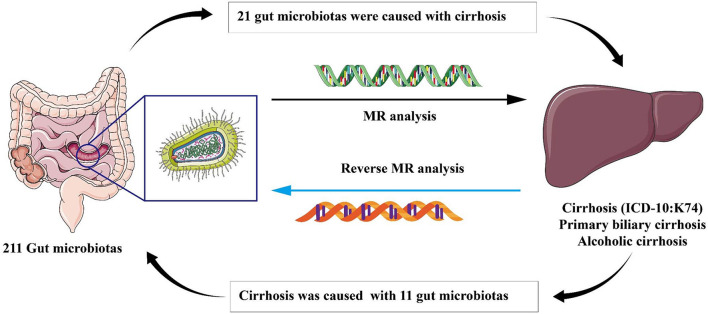

## 1. Introduction

In recent decades, studies have demonstrated that the entire gastrointestinal tract has a remarkable number of commensals with different functions in the pathophysiology of many intestinal and extraintestinal diseases (Tilg et al., 2022). Any perturbations between host and microbe interaction will upset the balance and cause diseases. Microbes and the liver mutually influence each other through metabolites and signals produced by dietary, genetic, and environmental factors, described as the Gut-Liver Axis (Albillos et al., 2020).

Cirrhosis is regarded as the end-stage of liver diseases and can cause portal hypertension and esophageal varices that lead to death (Tsochatzis et al., 2014). It is the 11th most common cause of death worldwide, and about 1 million people die every year due to cirrhosis (Gines et al., 2021). Many risk factors can lead to cirrhosis, including viruses, obesity, and alcohol consumption (Gines et al., 2021). However, a non-negligible factor is the microbes in the gastrointestinal tract (Tripathi et al., 2018). A complication of cirrhosis, portal hypertension, can damage the gastrointestinal mucosal barrier (Tilg et al., 2022). This damage unbalances the Gut-liver axis. The composition of intestinal flora changes, and then the secretion of inflammatory factors increases, which eventually leads to serious complications that have a significant impact on the treatment and prognosis of patients with liver cirrhosis (Chen et al., 2011; Bajaj et al., 2012; Sole et al., 2021). Gut flora is also a risk factor for hepatic encephalopathy (HE) in patients with cirrhosis (Vilstrup et al., 2014). However, current guidelines do not recommend the use of antibiotics or probiotics for the prevention of HE, especially in patients who have undergone trans-jugular intrahepatic portosystemic shunt (TIPS) (Gairing et al., 2022). This is because studies at this stage have produced conflicting results, and there is still a lack of large-scale randomized control trials (RCTs) to prove the benefit (Gairing et al., 2022). Furthermore, the gut microbiota is also susceptible to a variety of other factors, such as diet, the environment, and obesity (Tilg et al., 2022). Traditional methods, such as RCT, cannot solve the paradox of the interaction between bacteria and confounding factors by design, and thereby reduce the credibility of RCT (Vujkovic-Cvijin et al., 2020).

Mendelian randomization (MR) is a genetic epidemiological method that uses instrumental variables (IVs), strongly associated with exposure, to study causality and deal with confounding factors (Emdin et al., 2017; Davies et al., 2018). It is based on the fact that Single nucleotide polymorphisms (SNPs) are randomly variated and distributed during gamete formation, and it is not affected by confounding factors after gametogenesis. Since SNPs are not influenced by the outcome, we could exclude potential reverse causality effects (Bowden et al., 2015). MR, as a novel method, could address confounding factors and reverse causality by using genetic variants strongly associated with exposure to infer causality with outcome (Emdin et al., 2017; Davies et al., 2018).

In this study, we investigated the potential causal effect between 211 gut microbiota taxa and several subtypes of cirrhosis, including cirrhosis (ICD-10: K74), primary biliary cirrhosis (PBC), and alcoholic cirrhosis (ALC), by MR study design. Our study provided a new insight into the relationship between liver cirrhosis and gut microbiota.

## 2. Materials and methods

### 2.1. Study design

**Figure 1:**
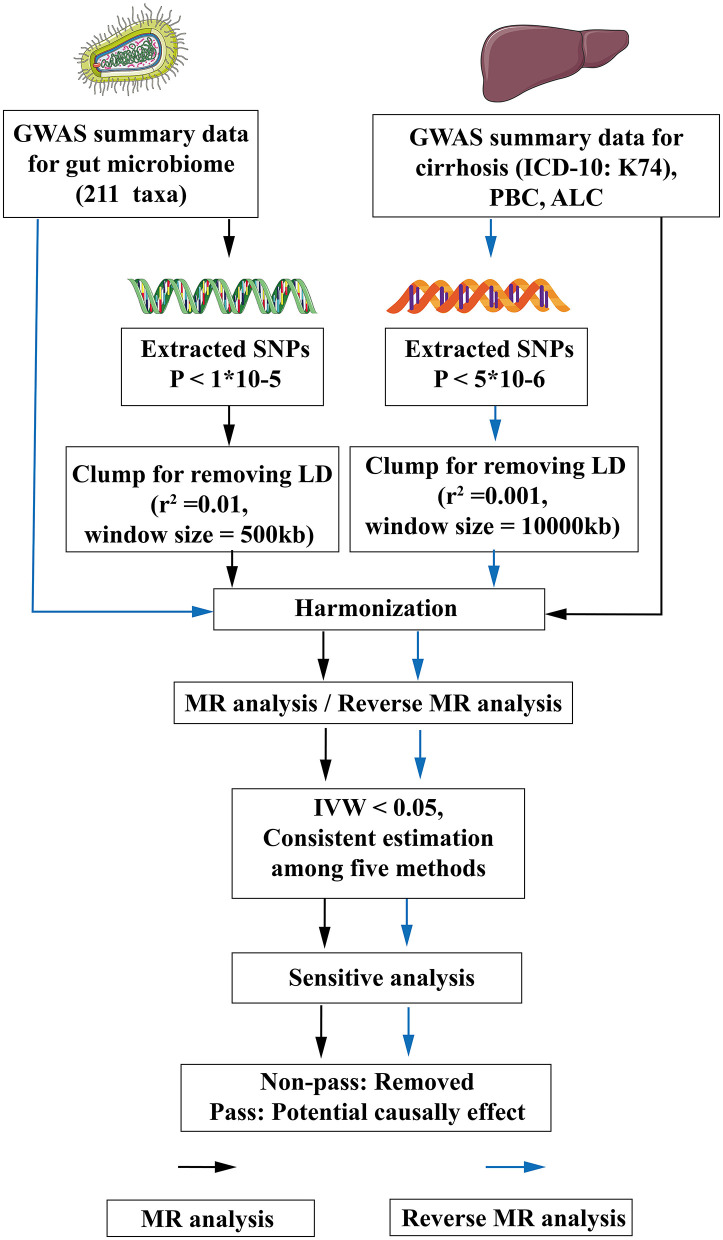
The flow chart of the study. The GWAS data of flora was used as exposure, and various subtypes of liver cirrhosis were used as outcomes for MR analysis. The instrumental variables of flora were extracted in the following way: (1) *P* < 1*10^−5^; (2) *r*^2^ = 0.01, kb = 500. Five methods were used for MR analysis after harmonization. When flora had an IVW < 0.05 and the estimated values of the five analytical methods were consistent, the flora were considered as significant, and then the pleiotropy and heterogeneity of the significant flora were detected. The pleiotropic and heterogeneous flora were discarded directly. Meaningful bacterial groups were screened out. Then reverse MR analysis was performed, and different criteria were used for the screening of instrumental variables for various subtypes of liver cirrhosis (*P* < 5*10^−6^, *r*^2^ = 0.001, kb = 10,000). MR, Mendelian randomization; LD, linkage disequilibrium; GWAS, Genome Wide Association Study; SNPs, single nucleotide polymorphisms; ICD-10, International Classification of diseases-10; IVW, inverse variance weighting; PBC, Primary biliary cirrhosis; ALC, Alcoholic cirrhosis.

For the study, 211 gut microbiota taxa were selected as exposure, and cirrhosis was defined as the outcome for MR analysis. Then, the exposure and outcome were exchanged for reverse MR analysis. All MR analysis in this study was executed under three basic assumptions: (1) IVs must be strongly correlated with exposure, (2) IVs cannot be correlated with confounding factors, and (3) IVs can only affect outcomes through exposure factors (Bowden and Holmes, 2019). The flow-chart of the study was shown in Figure 1.

### 2.2. GWAS data of gut microbiome

The large-scale GWAS summary data of gut microbiomes were obtained from the Mibiogen consortium, and included 18,340 individuals from 24 cohorts (Kurilshikov et al., 2021). This study used three different regions of the 16S rRNA gene to analyze the composition of gut microbiota and identified genetic variants that influence the relative abundance of microbial taxa by use of microbiota quantitative trait loci (mbQTL) mapping (Kurilshikov et al., 2021). Finally, 211 taxa were defined, namely 131 genera, 35 families, 20 orders, 16 classes, and 9 phyla.

### 2.3. GWAS data of cirrhosis

The GWAS summary data of cirrhosis (K74) and PBC were obtained from the FinnGen database. According to the International Classification of diseases-10 (ICD-10) diagnostic code (K74: Fibrosis and cirrhosis of liver, K74.3: Primary biliary cirrhosis), the patient was confirmed as having cirrhosis or PBC. The data were made up of 1,602 cases and 332,951 controls in the cirrhosis (K74) and included 497 cases and 257,081 controls in the PBC cohort (Kurki et al., 2023). All individuals included in the study were of European ancestry.

A GWAS meta-analysis data of ALC was acquired from Buch et al. (2015). Buch's study performed GWAS by use of 712 cases and 1,426 controls (discovery cohorts), and another two independent European cohorts (1,148 cases and 922 controls) validated the outcome of discovery cohorts (Buch et al., 2015). The case in Buch's study was defined as ALC by clinically diagnosed or biopsy-proven cirrhosis with a history of alcoholism and exclusion of other causes of cirrhosis. We selected the primary data as the exposure or outcome in our study. All individuals were of European ancestry. Detailed information of cirrhosis is available in Supplementary Table 1.

### 2.4. The selection of IVs

The criterion of selecting IVs is as follows: (1) SNPs, significantly associated with 211 gut microbiotas, were selected by the TwoSampleMR package of R software (the *P*-value of SNPs < 1^*^10^−5^) as the potential eligible IVs (Yu et al., 2023); (2) SNPs were clumped to exclude the effect of linkage disequilibrium (*r*^2^ = 0.01, window size = 500 kb; Xu et al., 2021); and (3) palindromic alleles were removed. Then, for the reverse MR analysis, we filtered the IVs of cirrhosis. However, *P* ≤ 5^*^10^−8^ is so strict that small SNPs were filtered out. Thus, we chose *P* ≤ 5^*^10^−6^ as the criterion and the clump was reset (*r*^2^ = 0.001, window size = 10,000 kb; Yu et al., 2023). Other criterions were the same as above. To avoid weak instrument bias, the F statistics for each bacterial taxon was calculated with the following equation (Xu et al., 2021):

$$\begin{array}{l} F=R^{2}\times \frac{n-1-k}{\left(1-R^{2}\right)\times k} \end{array}$$

In this equation, *R*^2^ is to explain exposure variance of the IVs, n is the sample size, and k is the number of IVs (Xu et al., 2021). According to a previous study, an F statistic ≥10 was considered as no weak instrument bias (Pierce et al., 2011).

### 2.5. MR analysis

We used five methods [namely Inverse variance weighted (IVW), MR Egger, Weighted median (WM), Simple mode, and Weighted mode method] to estimate the causal effect of the gut microbiota on cirrhosis. Due to the assumption that all instrumental variables are valid, IVW is susceptible to the effects of instrumental variable pleiotropy and heterogeneity. However, in the absence of these influences, IVW is considered to be the most accurate method, even when the other four methods do not yield positive results (Wang et al., 2023). Thus, the result of MR analysis was mainly based on IVW (Burgess et al., 2013). The other four methods were regarded as complements for IVW. After the IVs were matched, if the SNPs of the matching flora were < 3, we eliminated this microbiota, since the results for these bacterial groups were not credible. When the estimated values of the five methods were consistent and IVW < 0.05, the gut microbes were considered as having a significant difference.

### 2.6. Reverse MR analysis

To explore whether cirrhosis had a causal impact on the other gut microbiomes, we also performed a reverse MR analysis (cirrhosis as the exposure and gut microbiome as the outcome) using SNPs that are strongly associated with cirrhosis as IVs. The method of analysis was the same as MR analysis.

### 2.7. Analysis of horizontal pleiotropy and heterogeneity

The significant microbiotas were tested for pleiotropy and heterogeneity to ensure the accuracy of the IVW results. MR-Egger Intercept Test and Mendelian Randomization Pleiotropy RESidual Sum and Outlier (MR-PRESSO) global test were employed to detect horizontal pleiotropy (Long et al., 2023). MR-PRESSO could assess the overall horizontal pleiotropy of IVs and the abnormal SNPs which led to the pleiotropy (Verbanck et al., 2018). A *P*-value of two methods >0.05 showed that horizontal pleiotropy did not exist. To assess the degree of heterogeneity, Cochran's *Q* test was used and considered as non-heterogenous by *P* > 0.05. In accordance with the literature, in cases where heterogeneity is present (*P* < 0.05), the Inverse-Variance Weighted (IVW) method employed a random-effects model. Conversely, in the absence of heterogeneity, a fixed-effects model was utilized. After implementing the random-effects model, if heterogeneity persisted, the corresponding microbiota was excluded from the analysis (Papadimitriou et al., 2020). However, in instances where no heterogeneity was observed, the results of the random-effects model and fixed-effects model in IVW yielded consistent outcomes (Yuan et al., 2022). Hence, within our study, we calculated *P*-values utilizing the random-effects model in the IVW approach. Additionally, any microbiota exhibiting a Cochran's *Q* test *P*-value below 0.05 was subsequently omitted from subsequent analyses. This meticulous approach was undertaken to reinforce the robustness of results obtained through the IVW method. Meanwhile, Leave-one-out analysis was employed to exclude the influence of a single SNP.

### 2.8. Data processing

All data processing and analysis were accomplished by R software (R.4.2.3; http://www.R-project.org). The R packages used in the study are TwoSampleMR, MendelianRandomization, and MR-PRESSO.

## 3. Results

### 3.1. Instrumental variables for gut microbiome and cirrhosis

After strong correlation screening (*P* < 1^*^10^−5^) and clump (*r*^2^ = 0.01, window size = 500 kb), 2,895 instrumental variables were extracted from the gut microbiome (Supplementary Table 2). For reverse MR analysis, cirrhosis (K74) screened IVs based on *P* < 5^*^10^−6^ and clump (*r*^2^ = 0.001, window size = 10,000 kb), and finally 23 IVs (such as rs1430060, rs1882347, rs6800736) were screened for cirrhosis (K74), 16 IVs (such as rs11209051, rs113126523, rs11123970) were screened for PBC, and five IVs (such as rs62190923, rs6556045, rs7812374) for ALC (Supplementary Tables 3–5).

### 3.2. Causal effects of gut microbiome on cirrhosis risk by MR analysis

**Figure 2:**
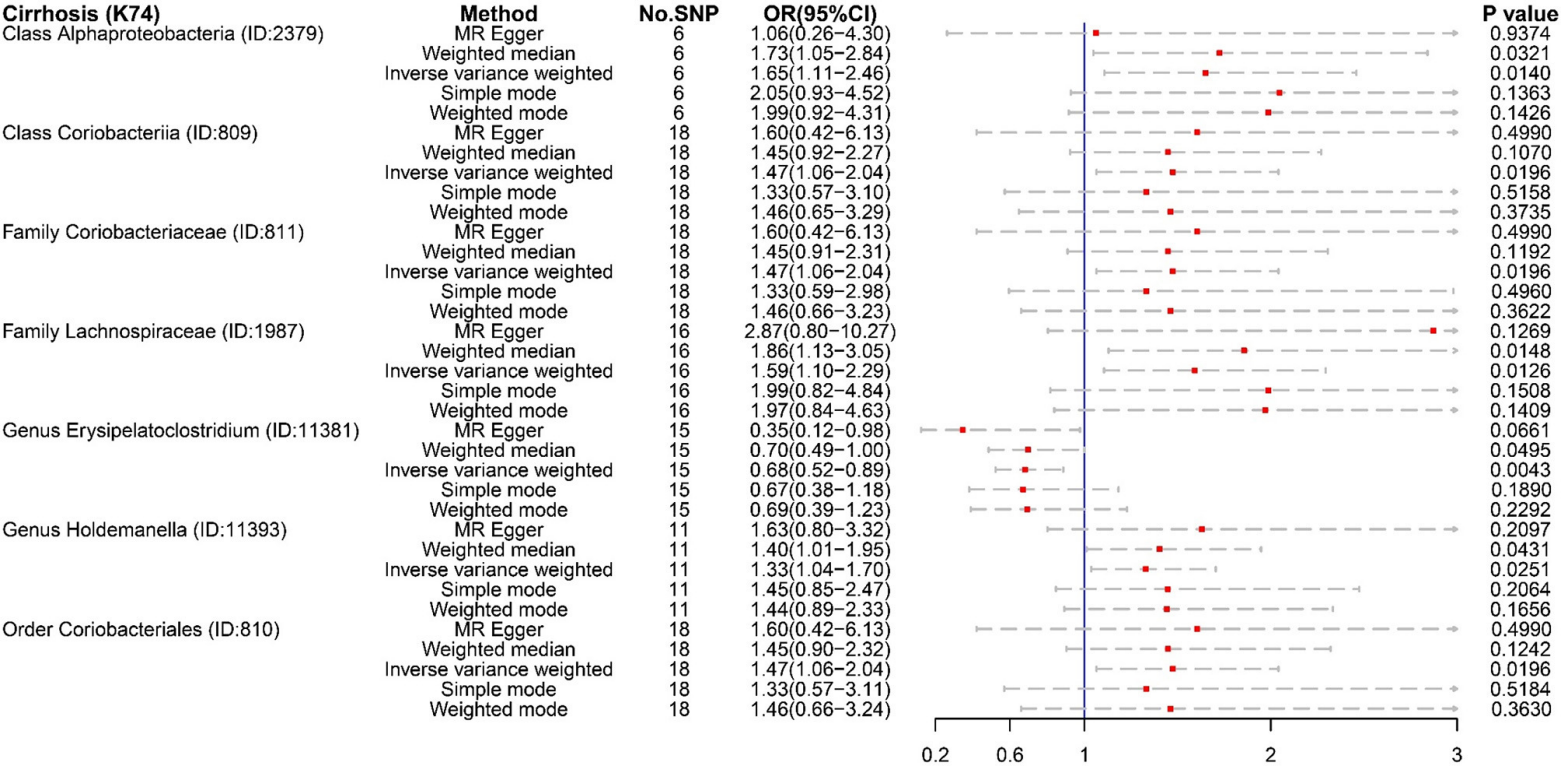
The result of MR analysis between gut microbiome and cirrhosis (K74) by five methods.

Several microbiotas displayed a potential causal effect on cirrhosis (Figure 2). The results showed that Class *Alphaproteobacteria* (OR:1.65, 95% CI:1.11–2.46), Class *Coriobacteriia* (OR:1.47, 95% CI:1.06–2.04), Family *Coriobacteriaceae* (OR:1.47, 95% CI:1.06–2.04), Family *Lachnospiraceae* (OR:1.59, 95% CI:1.10–2.29), Genus *Holdemanella* (OR:1.33, 95% CI:1.04–1.70), and Order *Coriobacteriales* (OR:1.47, 95% CI:1.06–2.04) were correlated with a higher risk of cirrhosis (K74). This suggests that these microbiotas might be risk factors for cirrhosis. Furthermore, Genus *Erysipelatoclostridium* (OR:0.68, 95% CI:0.52–0.89) might a protective factor for cirrhosis (K74).

**Figure 3:**
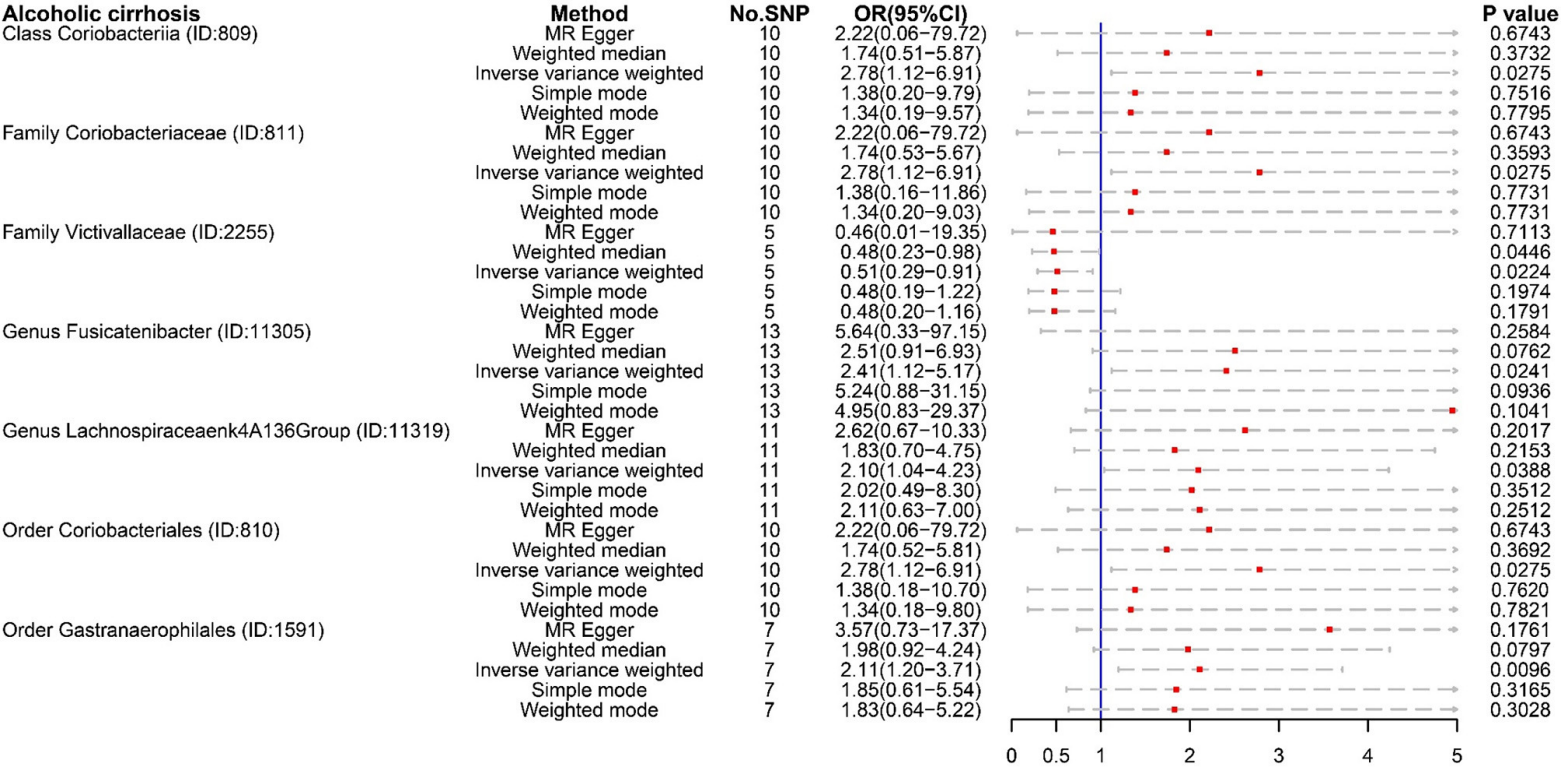
The result of MR analysis between gut microbiome and alcoholic cirrhosis by five methods.

For ALC, Class *Coriobacteriia* (OR:2.78, 95% CI:1.12–6.91), Family *Coriobacteriaceae* (OR:2.78, 95% CI:1.12–6.91), and Order *Coriobacteriales* (OR:2.78, 95% CI:1.12–6.91) were regarded as risk factors. These floras were also risk factors for cirrhosis (K74) (Figure 3). ALC had its own dangerous flora, such as Genus *Fusicatenibacter* (OR:2.41, 95% CI:1.12–5.17), Order *Gastranaerophilales* (OR:2.11, 95% CI:1.20–3.71), and Genus *Lachnospiraceae NK4A136 group* (OR:2.10, 95% CI:1.04–4.23). It was worth noting that Family *Victivallaceae* (OR:0.51, 97%CI: 0.29–0.91) was a potential protective factor for ALC. Compared with Family *Lachnospiraceae* in cirrhosis (K74), the Genus *Lachnospiraceae NK4A136 group* is more harmful.

**Figure 4:**
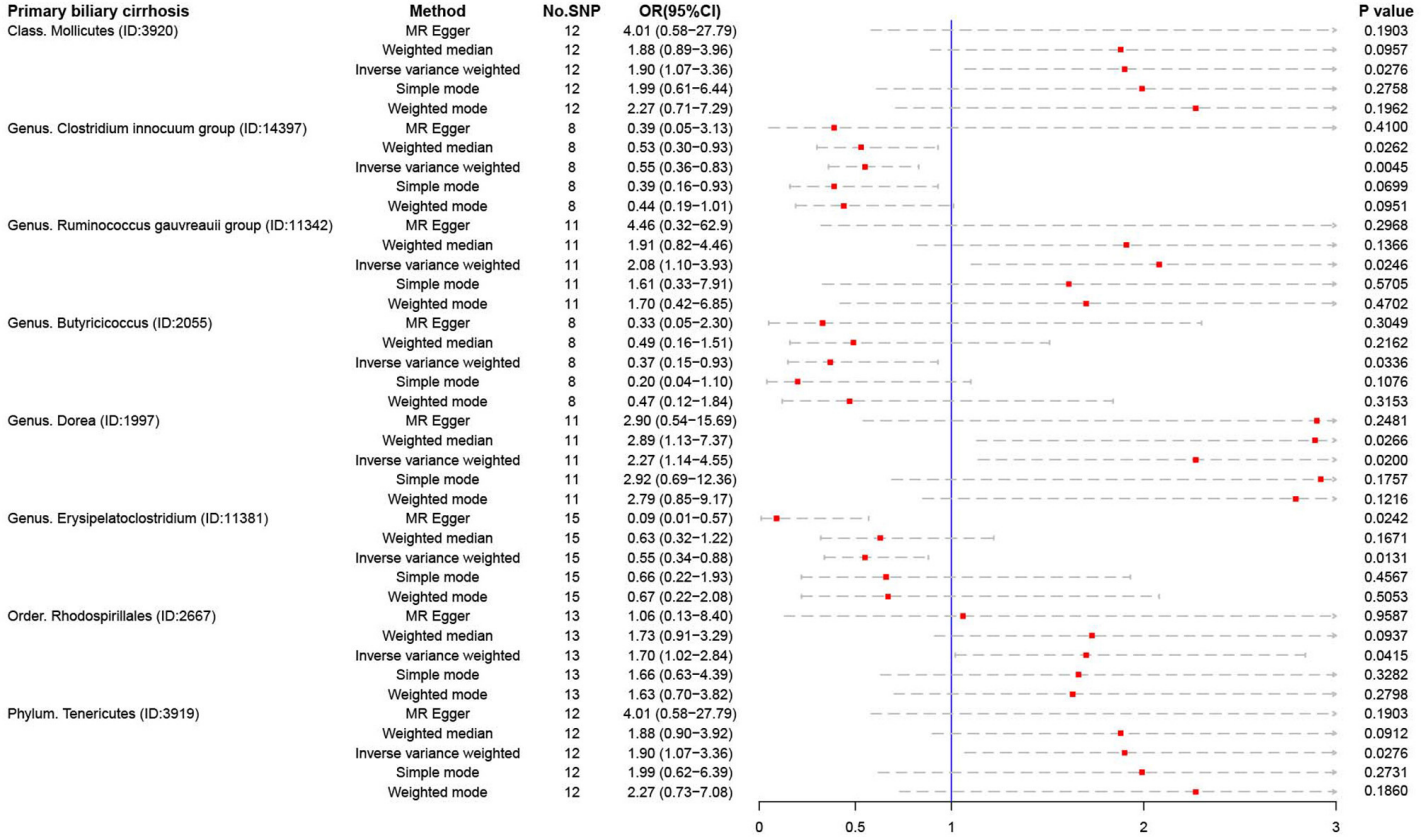
The result of MR analysis between gut microbiome and primary biliary cirrhosis by five methods.

For PBC, several microbiotas were associated with the occurrence of PBC (Figure 4), such as Class *Mollicutes* (OR:1.90, 95% CI:1.07–3.36), Genus *Ruminococcus gauvreauii* (OR:2.08, 95% CI:1.10–3.93), Genus *Dorea* (OR:2.27, 95% CI:1.14–4.55), Order *Rhodospirillales* (OR:1.70, 95% CI:1.02–2.84), and Phylum *Tenericutes* (OR:1.90, 95% CI:1.07–3.36). But Genus *Clostridium innocuum* (OR:0.55, 95% CI:0.36–0.83), Genus *Butyricicoccus* (OR:0.37, 95% CI:0.15–0.93), and Genus *Erysipelatoclostridium* (OR:0.55, 95% CI:0.34–0.88) were regarded as protective factors.

### 3.3. Causal effects of cirrhosis on gut microbiome by reverse MR analysis

**Figure 5:**
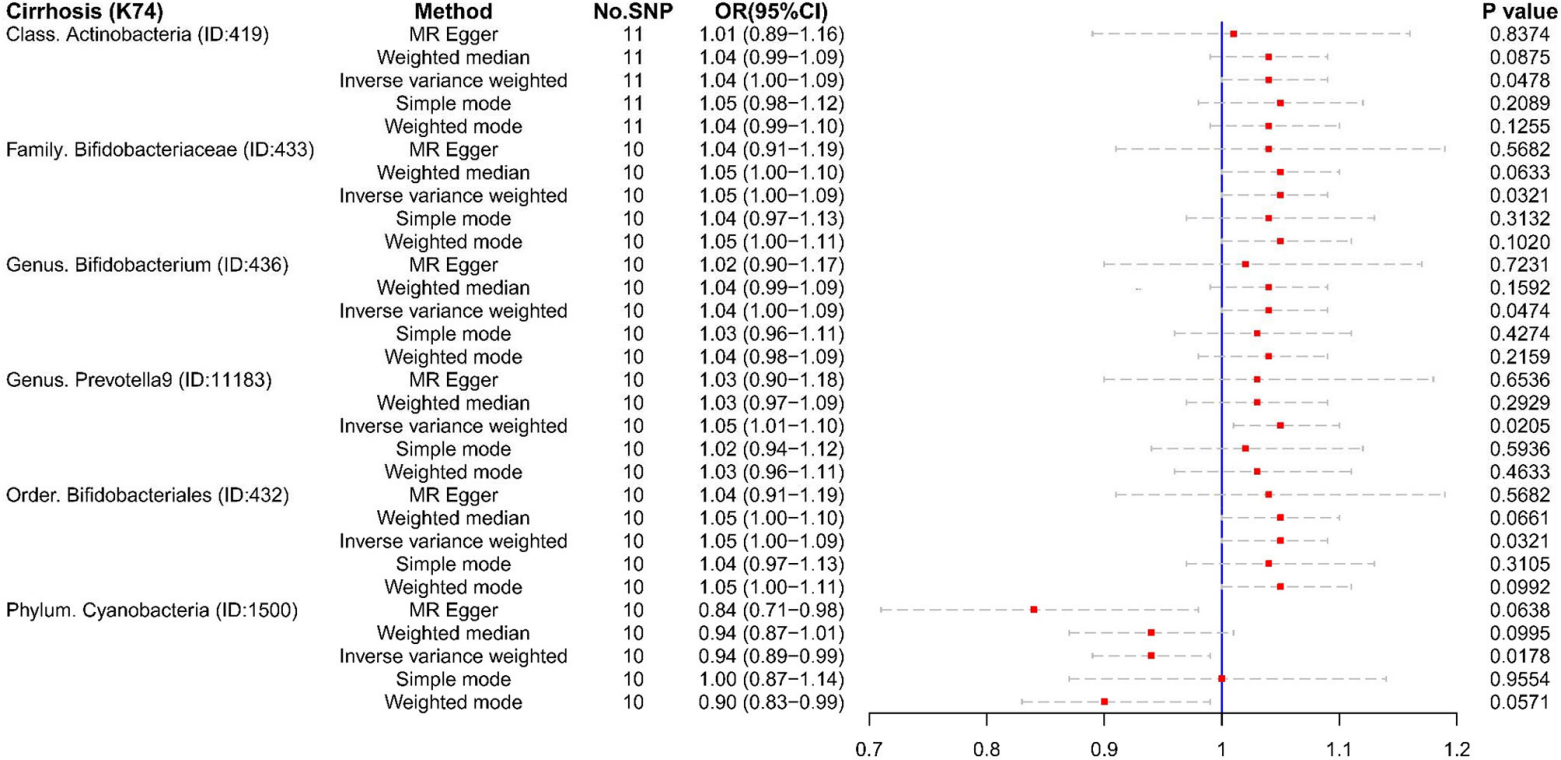
The result of reverse MR analysis between gut microbiome and cirrhosis (K74) by five methods.

In reverse MR analysis, we found four microbiotas were potential risk factors for Cirrhosis (K74): Class *Actinobacteria* (OR:1.04, 95% CI:1.00–1.09), Family *Bifidobacteriaceae* (OR:1.05, 95% CI:1.00–1.09), Genus *Bifidobacterium* (OR:1.04, 95% CI:1.00–1.09), Genus *Prevotella9* (OR:1.05, 95% CI:1.01–1.10), and Order *Bifidobacteriales* (OR:1.05, 95% CI:1.00-1.09). This suggested that the relative abundance of the above bacteria would decrease in the cirrhotic state. What iss interesting is that cirrhosis (K74) is a protective factor for Phylum *Cyanobacteria* (OR:0.94, 95% CI:0.89–0.99). Phylum *Cyanobacteria* may be more suitable for liver cirrhosis (Figure 5).

**Figure 6:**
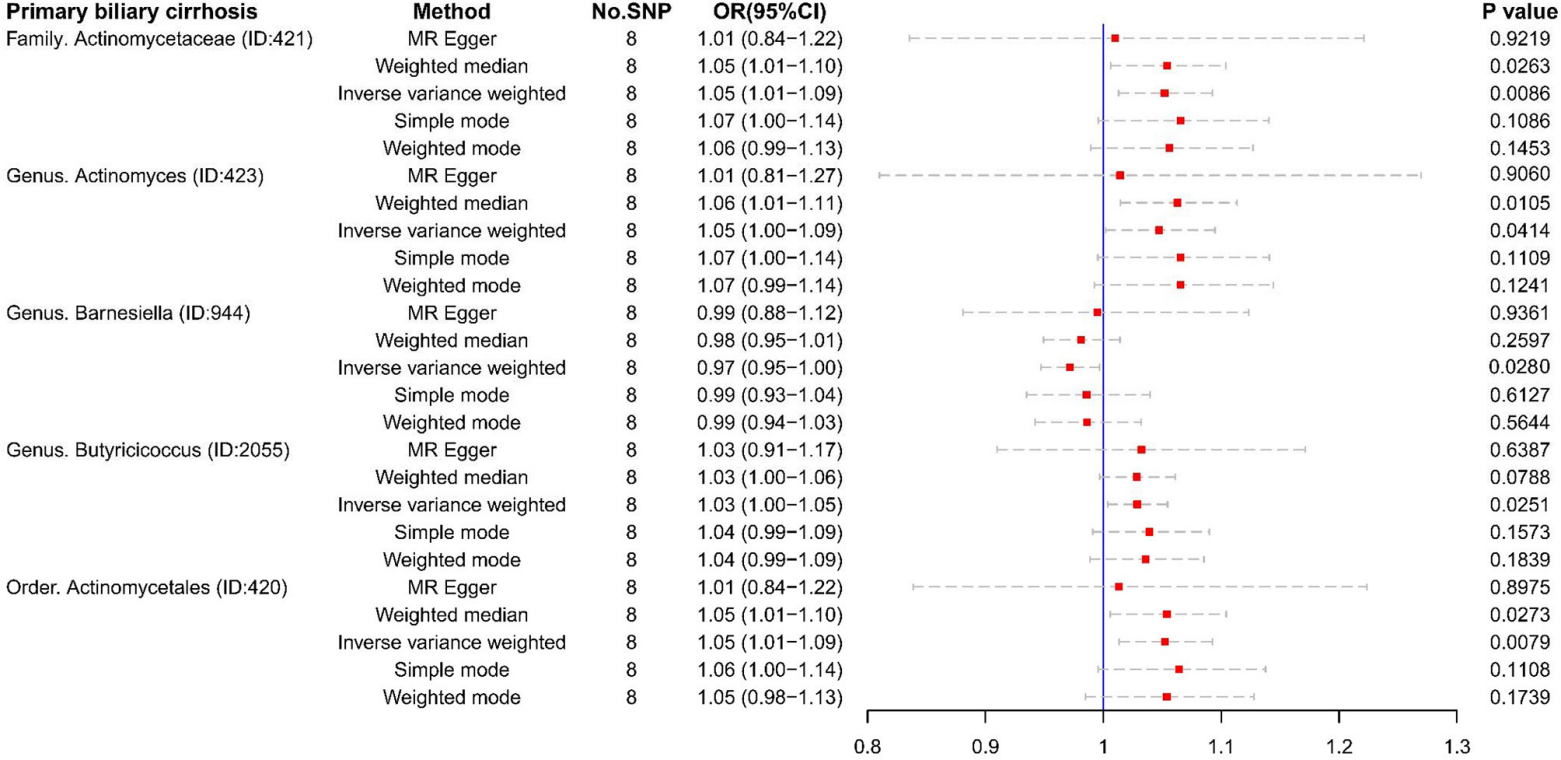
The result of reverse MR analysis between gut microbiome and primary biliary cirrhosis by five methods.

**Figure 7:**
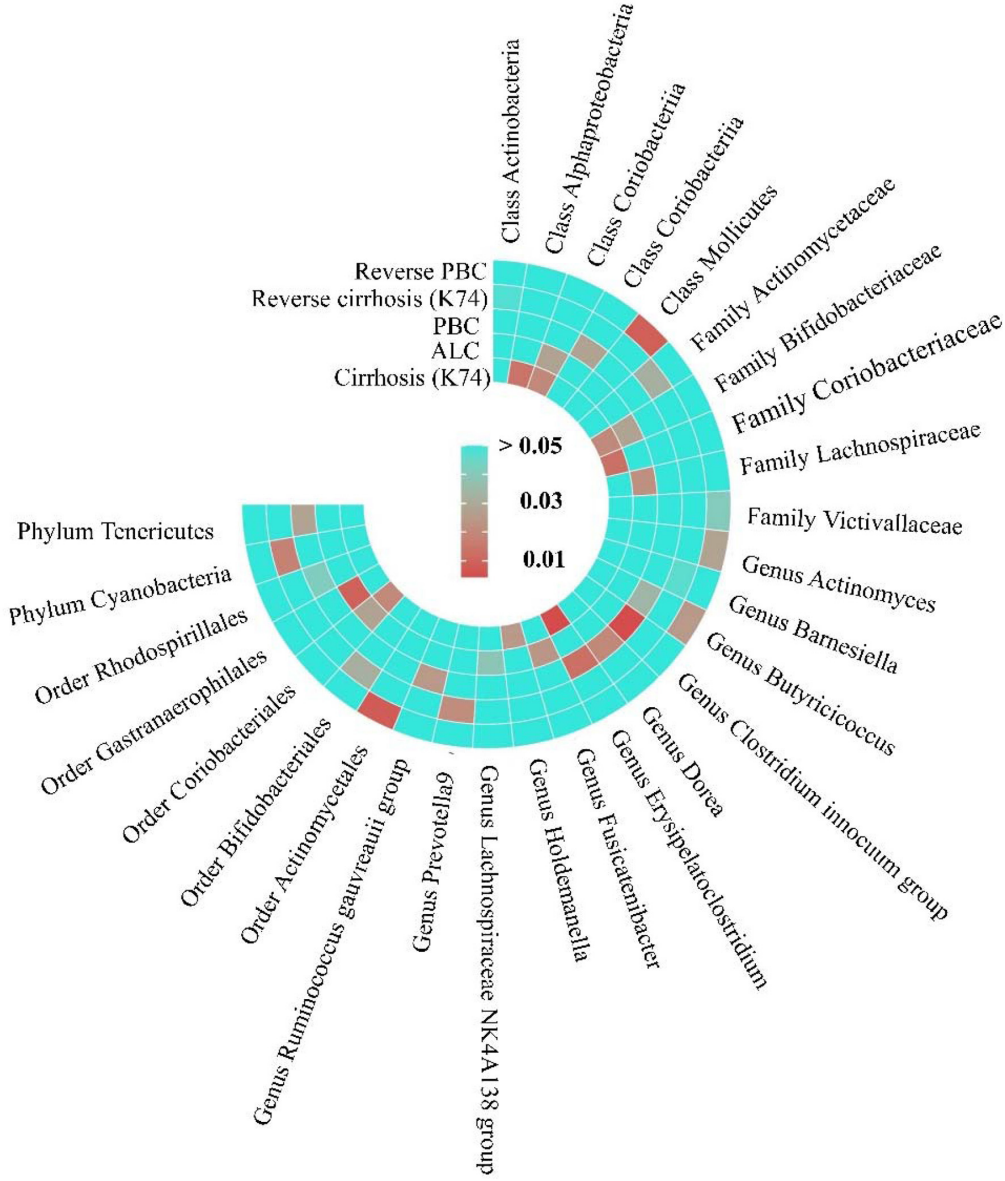
The Heatmap of MR analysis and Reverse MR analysis between gut microbiome and cirrhosis. This figure is based on the *P*-value of inverse variance weighting (IVW).

Unfortunately, there were too few SNPs after ALC matching to perform reverse MR analysis. For PBC (Figures 6, 7), Family *Actinomycetaceae* (OR:1.05, 95% CI:1.01–1.09), Genus *Actinomyces* (OR:1.05, 95% CI:1.00–1.09), Genus *Butyricicoccus* (OR:1.03, 95% CI:1.00–1.05), and Order *Actinomycetales* (OR:1.05, 95% CI:1.01–1.10) also had causal effects under the condition of PBC. But PBC was a potential protective factor for Genus *Barnesiella* (OR:0.97, 95% CI:0.95–1.00).

### 3.4. Sensitivity analysis of MR

**Figure 8:**
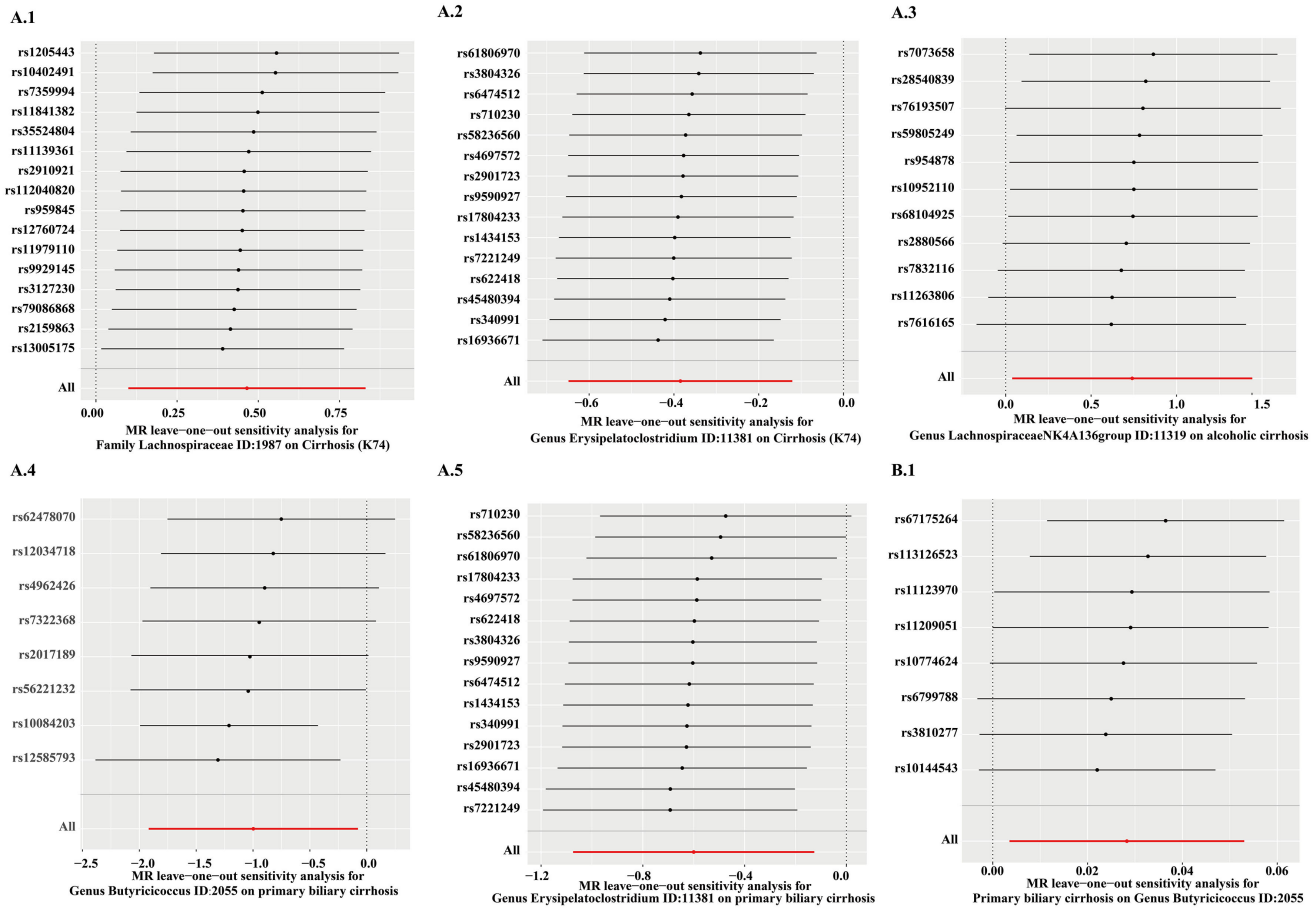
The results of leave-one-analysis in several gut microbiotas. MR analysis: **A.1**, Family *Lachnospiraceae;* **A.2**, Genus Erysipelatoclostridium; **A.3**, Genus *Lachnospiraceae NK4A136 group*; **A.4**, Genus *Butyricicoccus*; **A.5**, Genus *Erysipelatoclostridium*. Reverse MR analysis: **B.1**, Genus *Butyricicoccus*.

**Figure 9:**
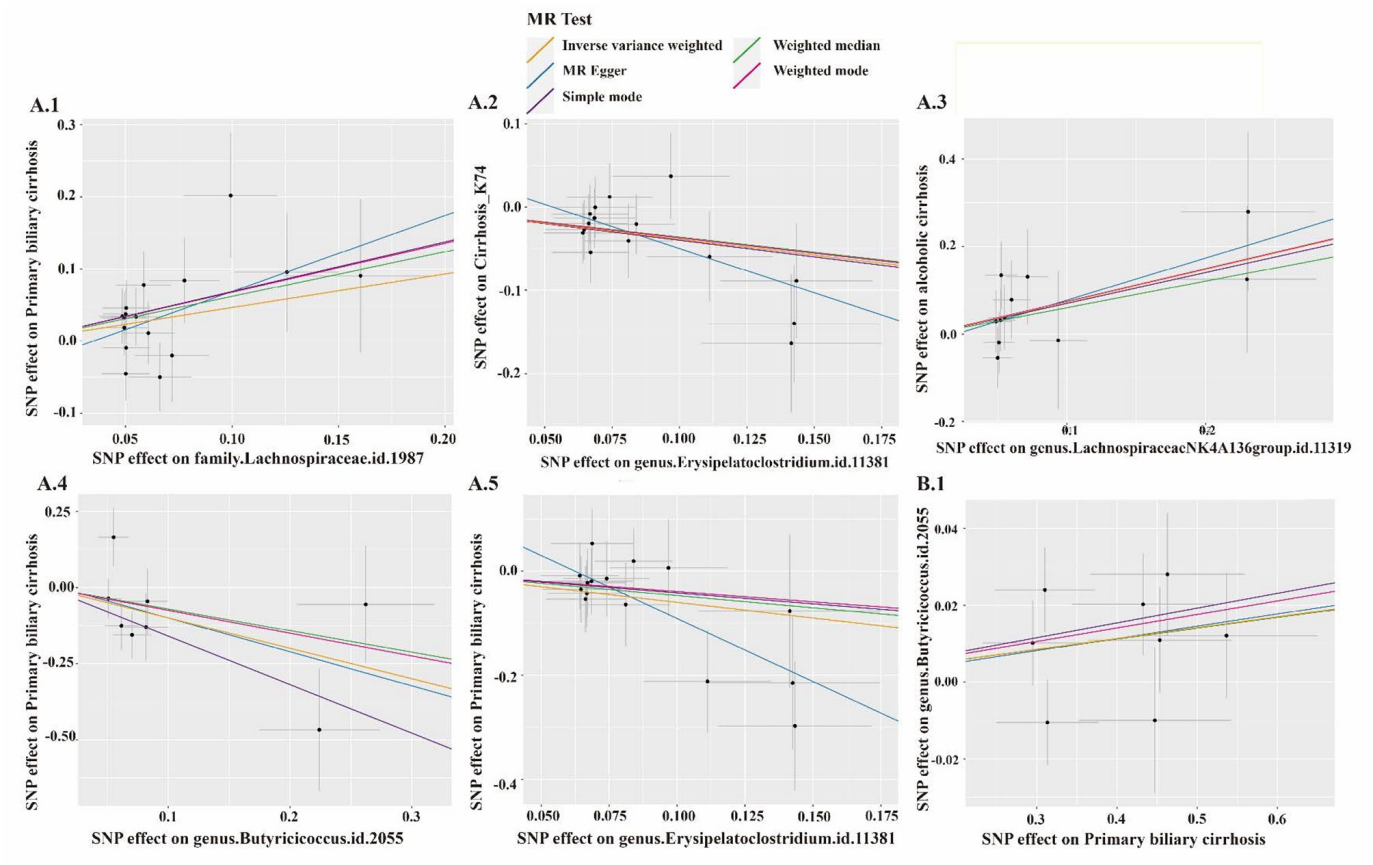
The scatter plot of several gut microbiotas. MR analysis: **A.1**, Family *Lachnospiraceae;* **A.2**, Genus Erysipelatoclostridium; **A.3**, Genus *Lachnospiraceae NK4A136 group*; **A.4**, Genus *Butyricicoccus*; **A.5**, Genus *Erysipelatoclostridium*. Reverse MR analysis: **B.1**, Genus *Butyricicoccus*.

MR PRESSO did not detect heterogeneity in significant floras (Tables 1, 2). The *P*-value of Cochran's *Q* test also showed no heterogeneity in the study (Tables 1, 2). Furthermore, the Leave-one-out analysis showed no significant different SNPs (Figure 8). In addition, no evidence of horizontal pleiotropy was detected by MR egger regression (*P* > 0.05) and all F statistical values were greater than 10 (Tables 1, 2). All the flora that passed the heterogeneity and sensitivity analysis met the following criteria, IVW was less than 0.05 and the estimates were consistent across the five methods (Figure 9).

**Table 1:** The results of pleiotropy and heterogeneity in MR analysis.

**Exposure**	**Outcome**	**MR-PRESSO global test**	**MR egger intercept test**	**Cochran's *Q* test**	***R* ^2^**	***F*-value**
*Class Alphaproteobacteria (ID:2379)*	Cirrhosis (K74)	0.65	0.55	0.67	0.02	23.07
*Class Coriobacteriia (ID:809)*		0.61	0.90	0.60	0.03	26.60
*Family Coriobacteriaceae (ID:811)*		0.61	0.90	0.60	0.03	26.60
*Family Lachnospiraceae (ID:1987)*		0.55	0.36	0.53	0.02	24.25
*Genus Erysipelatoclostridium (ID:11381)*		0.89	0.21	0.87	0.04	23.71
*Genus Holdemanella (ID:11393)*		0.99	0.56	0.98	0.05	27.49
*Order Coriobacteriales (ID:810)*		0.61	0.90	0.60	0.03	26.60
*Class Coriobacteriia (ID:809)*	ALC	0.84	0.90	0.83	0.01	21.97
*Family Coriobacteriaceae (ID:811)*		0.84	0.90	0.83	0.01	21.97
*Family Victivallaceae (ID:2255)*		0.97	0.96	0.97	0.04	24.74
*Genus Fusicatenibacter (ID:11305)*		0.81	0.56	0.77	0.02	23.69
*Genus Lachnospiraceae NK4A136 group (ID:11319)*		0.89	0.72	0.87	0.02	35.40
*Order Coriobacteriales (ID:810)*		0.84	0.90	0.83	0.01	21.97
*Order Gastranaerophilales (ID:1591)*		0.87	0.52	0.85	0.04	25.47
*Class Mollicutes (ID:3920)*	PBC	0.94	0.45	0.94	0.03	25.33
*Genus Clostridium innocuum group (ID:14397)*		0.65	0.75	0.61	0.05	22.54
*Genus Ruminococcus gauvreauii group (ID:11342)*		0.53	0.57	0.48	0.02	23.15
*Genus Butyricicoccus (ID:2055)*		0.23	0.90	0.18	0.02	39.72
*Genus Dorea (ID:1997)*		0.91	0.76	0.90	0.02	28.29
*Genus Erysipelatoclostridium (ID:11381)*		0.73	0.07	0.73	0.04	23.71
*Order Rhodospirillales (ID:2667)*		0.31	0.65	0.26	0.04	22.82
*Phylum Tenericutes (ID:3919)*		0.94	0.45	0.94	0.03	25.33

ALC, alcoholic cirrhosis; PBC, primary biliary cirrhosis.

**Table 2:** The results of pleiotropy and heterogeneity in reverse MR analysis.

**Exposure**	**Outcome**	**MR-PRESSO global test**	**MR egger intercept test**	**Cochran's *Q* test**	***R* ^2^**	***F*-value**
PBC	Family Actinomycetaceae (ID:421)	0.38	0.68	0.31	3.6^*^10^−4^ ~10^−3^	23.35~23.84
	Genus Actinomyces (ID:423)	0.19	0.78	0.14		
	Genus Barnesiella (ID:944)	0.87	0.71	0.84		
	Genus Butyricicoccus (ID:2055)	0.40	0.96	0.35		
	Order Actinomycetales (ID:420)	0.39	0.70	0.32		
Cirrhosis (K74)	Class Actinobacteria (ID:419)	0.20	0.67	0.14	7^*^10^−4^ ~10^−3^	31.07~39.33
	Family Bifidobacteriaceae (ID:433)	0.29	0.94	0.20		
	Genus Bifidobacterium (ID:436)	0.35	0.80	0.27		
	Genus Prevotella9 (ID:11183)	0.86	0.76	0.85		
	Order Bifidobacteriales (ID:432)	0.29	0.94	0.20		
	Phylum Cyanobacteria (ID:1500)	0.53	0.18	0.52		

PBC, primary biliary cirrhosis.

## 4. Discussion

In this study, we analyzed 211 microbiotas in the gut by bi-directional two sample MR analysis to detect the potential causal association between gut microbiota and cirrhosis. Previous literature suggested that *Lachnospiraceae* was beneficial for liver cirrhosis (Family *Lachnospiraceae*) (Chen et al., 2011; Bajaj et al., 2014, 2021). However, *Lachnospiraceae*, in our study, was considered as a risk factor for cirrhosis. This discovery was also shown in the process of exploring gut microbiota and ALC (Genus *Lachnospiraceae NK4A136 group*). Currently, no randomized controlled trials (RCTs) have directly substantiated the protective impact of *Lachnospiraceae* on liver cirrhosis. So far, only a change in the abundance of *Lachnospiraceae* has been detected prior to fecal transplantation (Bajaj et al., 2021). This observation implies that the indigenous microbiota may confer a safeguarding influence on *Lachnospiraceae*. However, a definitive deduction regarding *Lachnospiraceae* as a protective factor for liver cirrhosis cannot be established. Furthermore, it is noteworthy that *Lachnospiraceae* encompasses multiple distinct species that potentially assume diverse roles in the context of cirrhosis. In this regard, we put forward a hypothesis: *Lachnospiraceae* can lead to the occurrence of liver cirrhosis. In the state of cirrhosis, the proliferation rate of some intestinal flora was accelerated, resulting in the inhibition of the growth of *Lachnospiraceae*. We maintained that cirrhosis was not responsible for the inhibition of *Lachnospiraceae* growth, as this possibility was formally absent from the reverse MR analysis.

*Bifidobacteriaceae* are involved in the metabolism of short-chain fatty acids and bile acids; they were, thus, considered as beneficial bacteria (Ridlon et al., 2013; Riviere et al., 2016). In the reverse MR analysis, we found that cirrhosis (K74) is a risk factor for *Bifidobacteria* at the levels of Genus, Family, and Order (Family *Bifidobacteriaceae*, Genus *Bifidobacterium*, and Order *Bifidobacteriales*). We did not find a protective effect of *Bifidobacterium* on cirrhosis. For a long time, *Bifidobacterium* has been considered as a kind of beneficial bacteria for liver cirrhosis. An animal study revealed a marked decrease in the abundance of *Bifidobacterium* during the pathophysiology of fat-associated cirrhosis and liver cancer induced by high-cholesterol diet (Zhang et al., 2021). A clinical trial showed that *Bifidobacterium* can promote the transformation of macrophages and control the inflammatory response in patients with liver cirrhosis (Moratalla et al., 2016). However, our results showed that *Bifidobacterium* did not have a protective effect on liver cirrhosis but reduced in the cirrhotic state.

Furthermore, we found that Genus *Erysipelatoclostridium* was associated with a lower risk of cirrhosis (K74) and PBC. It has been observed that enrichment of *Faecalibacterium* was positively associated with *Erysipelatoclostridium* in healthy infants but not in cholestatic infants (Guo et al., 2018). Our findings suggested that there might be a direct effect of Genus *Erysipelatoclostridium* in improving the cirrhotic status of patients. Class *Coriobacteriia*, Family *Coriobacteriaceae*, and Order *Coriobacteriales* were regarded as risk factors for ALC in our study. These floras were the same as in the cirrhosis results (K74). However, there was no literature to report the causal effect of these microbiotas on cirrhosis. It is intriguing that *Coriobacterium* was considered a protective factor for type 2 diabetes due to its involvement in the metabolism of glutamate, but it was a risk factor for cirrhosis in our study (Zhuang et al., 2021). This indicates that the same microbiome plays different roles in different diseases.

In addition, it is worth noting that there are no reported studies on the causal effect of Genus *Butyricicoccus* on PBC. Distinctively, our study firstly demonstrated a bidirectional causal association between Genus *Butyricicoccus* and PBC. This association should be further investigated, and it may be a new target for PBC therapy.

Furthermore, some novel causal relationships were discovered in the present study. We found that Class *Alphaproteobacteria* and Genus *Holdemanella*, had a causal effect on the pathogenesis of cirrhosis (K74). There were several different taxa for ALC that could be risk factors, such as Genus *Fusicatenibacter*, Order *Gastranaerophilales*. Family *Victivallaceae* were a protective factor for ALC. In reverse MR analysis, Class *Actinobacteria* and Genus *Prevotella9* were inhibited in cirrhosis, but Phylum *Cyanobacteria* liked this environment. Some bacterial groups could cause the occurrence of PBC (Class *Mollicutes*, Genus *Ruminococcus gauvreauii*, Genus *Dorea*, Order *Rhodospirillales*, and Phylum *Tenericutes*). The causal relationship between PBC and these bacterial groups, however, was not observed in the reverse MR analysis. Genus *Clostridium innocuum* were beneficial for PBC in the MR analysis. In reverse MR analysis, Family *Actinomycetaceae*, Genus *Actinomyces*, and Order *Actinomycetales* were negatively affected under the condition of PBC, but Genus *Barnesiella* was positive under that. Further research is needed to confirm these causal relationships.

Our study has certain limitations. First, restricted by GWAS data, we can only accurately study the relationship between genus or above level and liver cirrhosis, but not validate a further specific level, such as species. Second, due to unavailable access to other GWAS data of ALC and the small size of IVs, we could not perform reverse MR analysis in ALC. Third, we did not analyze the relationship between viral cirrhosis, syphilitic cirrhosis, and other rare types of cirrhosis and gut microbiota. Fourth, we did not correct the *P*-value of IVW to control the Type I error rate. Due to the use of public GWAS data, the number of case and control groups in these cohorts was imbalanced, which may increase the likelihood of pleiotropy. Meanwhile, the effect of genetic mutations on other pathways may also affect the feasibility of the results. Finally, due to the lack of relevant GWAS data, we did not perform validation through replication samples.

In conclusion, we analyzed the causality between 211 gut microbiotas and cirrhosis. We demonstrated that 21 gut microbiotas might be risk or protective factors for cirrhosis, and 11 gut microbiotas might be influenced under the condition of cirrhosis.

## Data availability statement

The original contributions presented in the study are included in the article/Supplementary material, further inquiries can be directed to the corresponding authors.

## Author contributions

Q-AX: Formal analysis, Investigation, Methodology, Writing—original draft. Y-FY: Writing—original draft, Data curation. LC: Writing—original draft, Formal analysis. Y-CX: Investigation, Methodology, Software, Writing—review and editing. H-TL: Investigation, Methodology, Resources, Writing—review and editing. Z-GF: Investigation, Methodology, Software, Writing—review and editing. QH: Investigation, Methodology, Software, Writing—review and editing. JQ: Investigation, Methodology, Formal analysis, Writing—review and editing. JT: Formal analysis, Investigation, Data curation, Writing—review and editing. W-JZ: Data curation, Formal analysis, Investigation, Writing—review and editing. FC: Investigation, Methodology, Software, Writing—review and editing. Y-TH: Writing—review and editing. L-FA: Investigation, Resources, Software, Supervision, Writing—review and editing. CL: Resources, Software, Methodology, Visualization, Writing—review and editing. X-YC: Software, Formal analysis, Investigation, Writing—review and editing. DW: Supervision, Validation, Visualization, Writing—review and editing. Y-YT: Supervision, Validation, Visualization, Writing—review and editing, Project administration. XX: Project administration, Supervision, Validation, Visualization, Writing—review and editing, Funding acquisition, Resources. X-LZ: Funding acquisition, Project administration, Resources, Supervision, Validation, Visualization, Writing—review and editing.

## Supplementary material

Supplementary Figure 1 The causal effect between the gut microbiome and cirrhosis. The left half of the figure shows the forward Mendelian randomization results showing the causal effect of gut microbiota on cirrhosis. The right half of the figure shows the reverse Mendelian randomization results showing the causal effect of different types of cirrhosis on the gut microbiota. Risk factors are indicated in red and protective factors in dark green.

Supplementary Table 1 Overview of the source of cirrhosis data. This table is the information about the sources of GWAS data.

Supplementary Table 2 The instrumental variables of gut microbiome. The instrumental variables of gut microbiome were filtered under the condition that *P*-value is < 1^*^10^−5^, *r*^2^ = 0.01, and kb = 500.

Supplementary Table 3 The instrumental variables of Cirrhosis (K74). The instrumental variables of Cirrhosis (ICD-10: K74) were filtered under the condition that *P*-value is < 5^*^10^−6^, *r*^2^ = 0.001, and kb = 10,000.

Supplementary Table 4 The instrumental variables of Primary biliary cirrhosis. The instrumental variables of Primary biliary cirrhosis were filtered under the condition that *P*-value is < 5^*^10^−6^, *r*^2^ = 0.001, and kb = 10,000.

Supplementary Table 5 The instrumental variables of Alcoholic cirrhosis. The instrumental variables of Alcoholic cirrhosis were filtered under the condition that *P*-value is < 5^*^10^−6^, *r*^2^ = 0.001, and kb = 10,000.
