# Supplementary figures and images for: The causality between gut microbiome and liver cirrhosis: a bi-directional two-sample Mendelian randomization analysis
Source: Front Microbiol. 2023 Oct 18;14:1256874. doi: 10.3389/fmicb.2023.1256874 (PMC10619669; doi:10.3389/fmicb.2023.1256874)

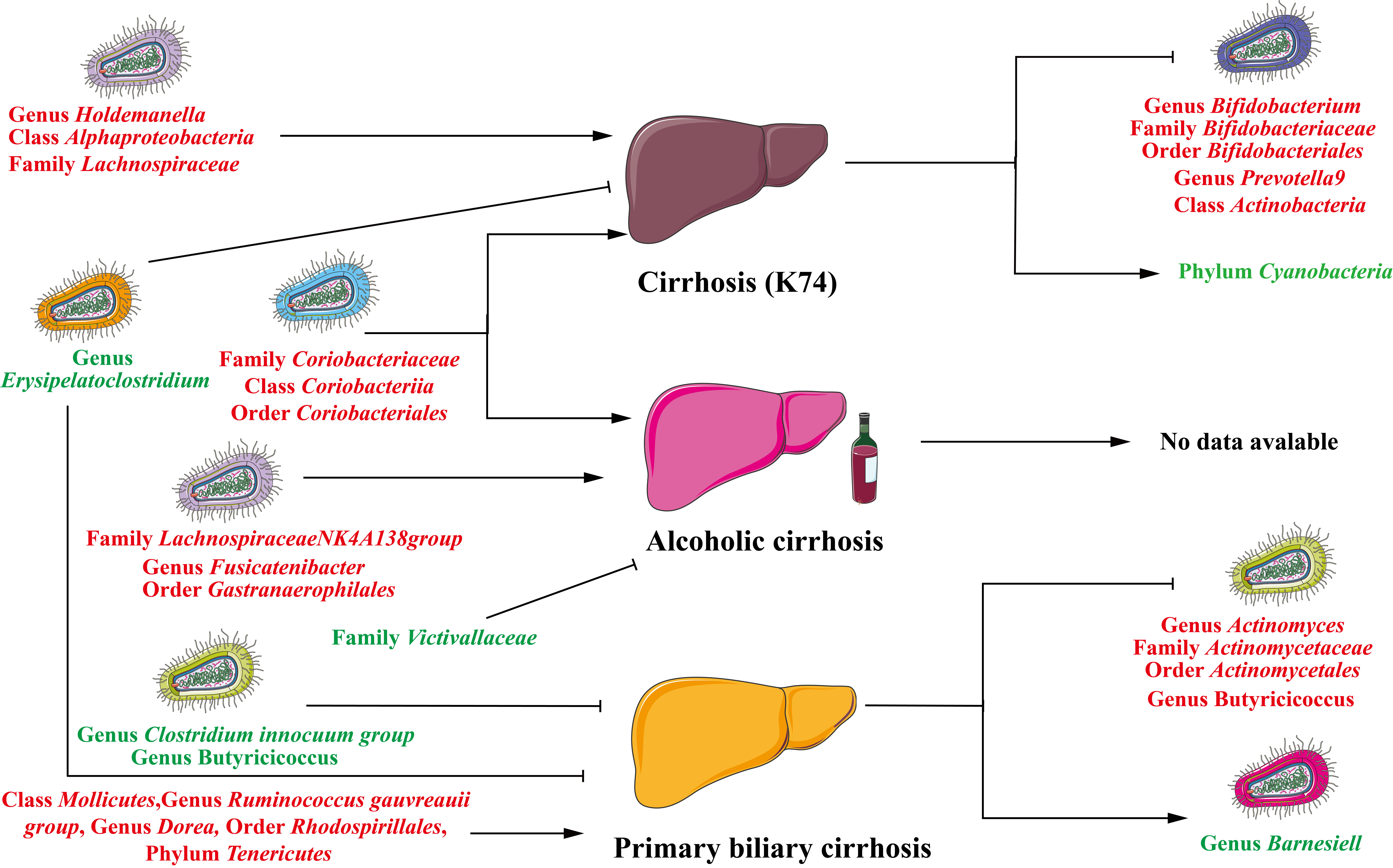

Supplement: Supplementary Figure 1 — The causal effect between the gut microbiome and cirrhosis. The left half of the figure shows the forward Mendelian randomization results showing the causal effect of gut microbiota on cirrhosis. The right half of the figure shows the reverse Mendelian randomization results showing the causal effect of different types of cirrhosis on the gut microbiota. Risk factors are indicated in red and protective factors in dark green. [file Image_1.JPEG]
